# Extracellular vesicles derived from different sources of mesenchymal stem cells: therapeutic effects and translational potential

**DOI:** 10.1186/s13578-020-00427-x

**Published:** 2020-05-24

**Authors:** Jiaxin Cai, Junyong Wu, Jiemin Wang, Yongjiang Li, Xiongbin Hu, Shifu Luo, Daxiong Xiang

**Affiliations:** 1 Department of Pharmacy, The Second Xiangya Hospital, Central South University, Furong District, Changsha, Hunan China; 2 Hunan Provincial Engineering Research Centre of Translational Medicine and Innovative Drug, Changsha, Hunan China; 3 Institute of Clinical Pharmacy, Central South University, Changsha, Hunan China; 4 Xiangya School of Pharmaceutical Sciences, Central South University, Changsha, China

**Keywords:** Extracellular vesicles, Exosomes, Mesenchymal stem cells

## Abstract

Mesenchymal stem cells (MSCs) were known to have excellent properties in cell therapy. However, the risk of immune rejection associated with cell transplant therapy hampers its use. Extracellular vesicles secreted by MSCs derived from different sources that contain therapeutic molecules such as RNA and proteins, which is a novel strategy for cell-free therapy. Recently, researches show EVs from MSCs (MSC-EVs) of different sources have special functions and effects on different diseases. Here, we collected these researches and compared them to each other. In addition, their potential and possible application in clinical treatment are described.

## Introduction

Mesenchymal stem cells(MSCs)are kinds of stem cells that play an important role in regeneration and restoration because of its multi-lineage differentiative capacity. They have the potential to differentiate to form connective tissues, skeletal muscle cells, and cells of the vascular system [1]. MSCs are developed from the mesodermal germ layer, which is one of the three layers in the inner mass cell [2]. There are a large number of MSCs in the parts of human bodies with high regeneration and differentiation capacity,such as bone marrow, embryo. Besides, the human umbilical cord and adipose tissue are also important origins of MSC for researches. The above four sources are the main sources of MSC researches and are also the main kinds we are going to introduce in this review. In addition to this, there are some rare sources of MSCs, including menstrual blood-derived MSCs [3, 4] and dental MSCs [5]. What they secreted have characteristics of MSCs and the potential for treatments of related diseases. Moreover, it has been discovered that MSCs also played an important role in immune regulation and might be a mediator of inflammation. The signals of inflammation activate MSCs to differentiate proinflammatory and anti-inflammatory phenotype and MSCs can also affect the polarization of monocytes and control pathogenic T cell responses [6, 7]. More recently, it was reported that MSCs transplanted into human bodies for treatments and received good effects. Thus, A new treatment called cell-therapy has emerged and developed [8]. Although MSCs transplantation seems to have a promising prospect for development, most clinical trials remained in phase I or II. And clinical trial failures of autologous and allogeneic MSC products have been frequent. There are even reports about the risk of tumorigenicity and cell death resulted from the transplantation of MSCs [9].

**Fig. 1:**
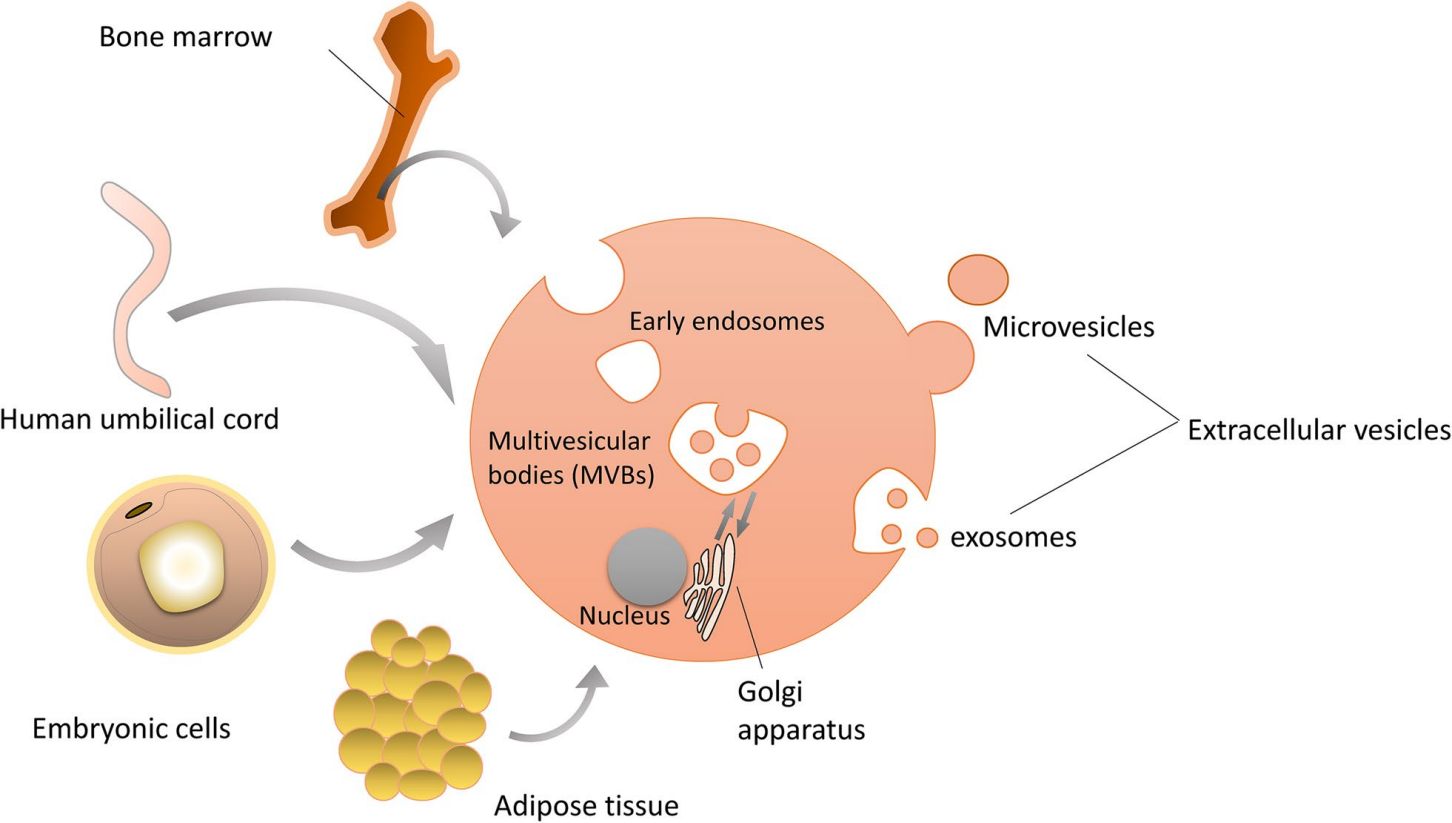
MSCs derived from different sources and EVs secreted. MSCs are mainly extracted from bone marrow, human umbilical cord, embryonic cells, and adipose tissue. And they secrete EVs such as microvesicles and exosomes

It has been shown that the therapeutic functions of MSCs mediated partly through paracrine effects. The various bioactive molecules that MSCs secreted can modulate immune, inhibit apoptosis and fibrosis, promote angiogenesis and the growth of stem and progenitor cells [10]. Some of them are mediated by extracellular vesicles (EVs), which were a series of vesicles secreted by MSCs (Fig. 1). EVs are cell-derived membranous structures that originate from the endosomal system or which are shed from the plasma membrane [11]. EVs are not a homogeneous system, including exosomes, shedding vesicles, apoptotic bodies, melanosomes, and prostasomes range from 10 to 1000 nm [12, 13]. Most researches mainly focused on exosomes and microvesicles (MVs). MVs are vesicles of which the particle size is > 200 nm, budding directly from the plasma membrane [14]. Exosomes have the smaller particle size that ranges from 50 nm to 200 nm, for they are formed through the invagination of the early endosome [15]. Unlike microvesicles, exosomes are cup-like vesicles with CD81, CD9, and Alix as their biomarkers, due to the different mechanisms of secretion [1, 16]. Exosomes also have some special biological characteristics and processes. Apart from those proteins that serve as biomarkers, exosomes also carry many functional cytokines and growth factors, regulatory RNAs and so on [15]. The release of exosomes is also a special process. It involves some proteins, such as the ESCRT [17]. The function of exosomes that attract much attention. In the beginning, exosomes were regarded as metabolic waste [18]. With more and more studies of exosomes, the functions of signal transmission and cell-to-cell communication were discovered [19]. On the one hand, they appear similar functions of MSCs and is expected to become the alternatives to cell therapies. On the other hand, it has been discovered that exosomes might influence the tumor microenvironment, but the mechanism remained unknown [20]. Thus, exosomes have the potential to treat cancers by changing the microenvironment of tumors.

In addition, EVs from different MSCs may show a unique tendency to the therapy of some diseases. And they appeared differently in the same diseases [4, 21]. There are several investigations showed the potential therapeutic effects in diseases of cardiac injury, kidney injury, and brain injury [12]. Recently, MSC-derived EVs as a cell-free therapeutic alternative have raised considerable interests. Compared to MSCs, they reduced the risk of cell transplantation and amplification. At the same time, they have the advantages of good stability. Not only that, but EVs can also be used as a vehicle to deliver bioactive factors. Moreover, EVs were proved to have the ability to cross the blood–brain barrier [22]. Considered these advantageous properties of the MSC-EVs, further studies need to be done.

In this review, we mainly compared the therapeutic effects of four different extracellular vesicles of mesenchymal stem cells (MSCs-EVs) and discussed their potential applications in diseases which include that they act as carriers of active biological molecules.

## EVs from bone marrow mesenchymal stem cells (BM-MSCs)

### The therapeutic effects of EVs from BM-MSCs

Extracellular vesicles derived from bone marrow mesenchymal stem cells is the most common one. And it is also a conventional way to obtain the MSCs-EVs, which is to harvest bone marrow from adult rats or humans [23, 24]. For extracellular vesicles from human bone marrow mesenchymal stem cells (hBM-MSCs), the most common diseases treated are cartilage defects or osteoarthritis which are related to bone diseases [25] [26]. The reason is that the bone marrow source has the bone targeting ability [27] and can also induce osteogenic differentiation similar to its parent cells [28, 29]. In a study of osteoarthritis, EVs from hBM-MSCs can promote extracorporeal cartilage regeneration because EVs stimulated chondrocytes to produce proteoglycans and type II collagen. Also, those EVs inhibit the adverse effects of inflammatory mediators on cartilage homeostasis. These anti-inflammatory effects were mediated by inhibiting TNF-alpha-mediated upregulation of COX2 and pro-inflammatory interleukins [26]. Since the bone marrow from which hMSC comes is connective tissue, the EVs they secreted were discovered to have a therapeutic effect on other connective tissue such as tendon. Topical administration of MSC-EVs promotes tendon healing by inhibiting the accumulation of inflammatory and apoptotic cells and increasing the proportion of tendon resident stem cells/progenitor cells [30].

EVs from hBM-MSCs have also shown good efficacy in the treatment of the renal injury. For chronic kidney injury caused by long-term exposure to heavy metals such as cadmium, Intravenous injection of high-purity EVs from hBM-MSCs repaired damage to renal proximal tubules, glomerular podocytes, bone deformation, and improved survival [31]. And in acute kidney injuries, EVs have a similar effect to parent cells in promoting kidney regeneration [32]. For diabetic nephropathy, EVs inhibit renal fibrosis and result in the downregulation of several fibrogenic genes in renal tissue [33].

In the aspect of anti-inflammation, EVs from hBM-MSCs works in several different ways. First, they downregulated the production of IL-23 and IL-22 and enhanced the anti-inflammatory phenotype of mature human regulatory macrophages (Mregs) [34]. Second, as mentioned in osteoarthritis treatment, they inhibited TNF-alpha-induced expression of COX2 and expression of pro-inflammatory interleukins [26].

Another application of EVs from hBM-MSCs is Graft-Versus-Host Disease Amelioration. Systemic infusion of hBM-MSCs EVs could prolong the survival time of aGVHD mice and reduce pathological damage of various GVHD-targeted organs. This was due to the immunosuppressive effect of hBM-MSCs derived extracellular vesicles. CD4 + and CD8 + T cells were inhibited in EV-treated GVHD mice. While inhibiting the functional differentiation of T cells from naive to effector type, EVs derived from BM-MSCs also retained the CD4 + CD25 + Foxp3 + regulatory T cell (Tregs) population [35]. EVs can significantly reduce the risk of cell transplantation while retaining the similar efficacy of BM-MSCs. In another study about the immunoregulatory effects of hBM-MSCs EVs, they induced the apoptosis of CD3 + cells and of the CD4 + subpopulation and increased the proliferation and the apoptosis of Tregs. In addition, EVs treatment increased the Treg/Teff ratio and the concentration of the immunosuppressive cytokine IL-10. These results suggest EVs have an immunomodulatory effect on T cells in vitro, but this is not related to IDO, which is an established mediator of MSC immunosuppressive effects [36].

Compared to EVs from hMSC, mouse bone marrow derived MSC-EVs have similar functions in tissue regeneration [37], protective effects to neuron cells [38] and irradiated hematopoietic stem cells [39]. However, it has been discovered that EVs from human MSCs (hMSCs) are generally more effective than those from mouse MSCs (mMSCs) in a study of allergic airway inflammation in the mouse model. Moreover, blocking the release of EVs completely abrogated the effects of hMSCs but only partially inhibited the effects of mMSCs, suggesting different potential mechanisms of mMSC versus hMSC actions [40].

### The therapeutic effects of exosomes from BM-MSCs

Exosomes are one type of extracellular vesicles, which size is smaller than others, for about 100 nm [17]. They have similar therapeutic effects to BM-MSCs and are expected to develop into a promising cell-free therapeutic strategy. In some studies, researchers investigated the potential mechanisms of human bone marrow mesenchymal stem cell derived exosomes (hBM-MSCs-Ex) in the treatment of liver fibrosis and found the therapeutic effect of exosomes on hepatic fibrosis was significantly greater than that of hBM-MSCs [41]. Moreover, recent studies have discovered that exosomes are the main mediators of MSC therapy in Autism Spectrum Disorder (ASD). In the previous study, transplanting human bone marrow mesenchymal stem cells (MSC) into the lateral ventricle of BTBR mice can improve their autistic phenotype for a long time. However, the nasal administration of MSC exosomes to BTBR mice can significantly improve autistic behavior that increased social interaction and reduced repetitive behaviors. This treatment can avoid the risk of rejection and infection associated with the transplant [42]. The exosomes, which are small size vesicles with strong permeability, are also very suitable for nasal administration. In addition, the role of hBM-MSCs-Ex in immunotherapy should not be ignored. Apart from inducing polarization of M2 macrophages, it also regulated the maturation, proliferation, and activation of lymphocytes. For example, the exosomes regulated the proliferation of T and B lymphocytes and affected the function of B lymphocytes by regulating the differential expression of related mRNAs [43].

More effects were investigated in mouse bone marrow MSCs derived exosomes. For inducing macrophage polarization, exosomes derived from mBM-MSCs have the same effect. In a study of wound healing, hBM-MSCs exosomes mainly regulated the polarization of macrophages by targeting pknox1 with mir-223 in exosomes [44]. However,in another study of heart attacks, LPS-primed mouse BMSC-derived exosomes had a good therapeutic effect on polarized M2 macrophages in vitro and reduce inflammation and myocardial cell apoptosis after infarction [45]. Similar to macrophage polarization, recent studies found that mouse BMSC-derived exosomes can regulate microglia polarization. The exosomes reduced inflammation and demyelination of the central nervous system in EAE rat models by increasing microglial cell M2 polarization [46]. In some studies of myocardial infarction, BMSC-derived exosomes showed therapeutic potential in improving myocardial function. GATA-4 is a cardiomyocyte-specific zinc finger transcription factor that regulates differentiation, growth, and survival. GATA-4-expressing BMSCs improve functional recovery than that observed for BMSCs alone when injected into the myocardium immediately after induction of ischemia. It was found that GATA-4-expressing mBM-MSCs exosomes can induce MSCs to differentiate into precursor cells of cardiomyocytes, reduce apoptosis of cardiomyocytes, and improve cardiac function after myocardial infarction [47]. Additionally, they significantly reduced the apoptosis rate of cardiac stem cells (CSCs) and the production of reactive oxygen species (ROS) after oxidative stress injury. And exosomes of hypoxic-cultured BM-MSCs were more potent than exosomes of normal cultured MSCs. Further researches showed that mir-214 is the main effector molecule in exosomes of BM-MSCs that protects CSCs from oxidative damage. BMSCs inhibited oxidative stress damage in CSCs by silencing the release of mir-214-containing exosomes by CaMKII [48]. Another study explains this phenomenon from another angle. Hypoxia culture enhanced the activity of mir-210 and nSMase2 in MSC and its secreted exosomes, which are important to the secretory pathway of exosomes [49].

### The transferring function of EVs from BM-MSCs

As vesicles secreted by cells, the contents of EVs have something in common with BM-MSCs, such as RNA. Some specific microRNAs secreted by BM-MSCs regulate cells function through EVs transportation. For example, EVs derived from BM-MSCs contains miR-146a which is a famous anti-inflammatory microRNA. Studies about Inflammatory bowel disease (IBD) showed that overexpressing miR-146a inhibited TNF receptor-associated factor 6 (TRAF6) and IL-1 receptor-associated kinase 1 (IRAK1) expression in TNBS-induced colitis of rats. And the administration of EVs containing miR-146a down-regulated the increased phosphorylation levels of NF-κB p65 and IκBα, so that suppressed inflammation factors such as tumor necrosis factor-α (TNF-α), Interleukin-6 (IL-6) and Interleukin-1β [50].

For exosomes, a type of smaller and more homogeneous extracellular vesicles, studies on microRNAs associated with them have been more extensive. At present, many microRNAs contained in exosomes have been revealed to associate with organ repair and other functions. It has been reported that low levels of miR-224-3p in BM-MSCs derived exosomes promote endothelial cell proliferation, migration, invasion, and angiogenesis by targeting focal adhesion kinase family interacting proteins (FIP200) [51]. In a study of renal ischemia/reperfusion injury, researchers found that miR-199a-3p enriched in hBMSC-Exos and down-regulate Sema3A expression, thereby activating the AKT and ERK pathways when delivering to renal cells by exosomes [52]. Mir -199a-5p was another microRNA that has been showed to protect renal ischemia/reperfusion injury. It is transferred to renal tubular epithelial cells (nrk-52e) in a time-dependent manner and significantly inhibits I/r-induced er stress by targeting immunoglobulin (BIP) [53]. Besides, other microRNAs that BMSCs-Exo contained related to other functions have also been studied. For example, the release of exosomes from BM-MSCs in elderly mice can be absorbed by fat cells, muscle cells and liver cells, resulting in insulin resistance in vivo and in vitro, which was related to miR-29b-3p. Down-regulation of it significantly improved insulin resistance in older mice [54].

In addition to the transport of endogenous RNA, BM-MSCs EVs can also act as carriers for the transport of other small molecules. Because of its own therapeutic effect on some diseases or targeting ability or greater affinity to specific cells, it may be suitable for EVs to transport therapeutic components. As one type of EVs, microvesicles were found to be endogenous carriers for delivering paclitaxel to pancreatic cancer cells. Microvesicles capsuled Paclitaxel proved to have strong anti-proliferation activity on pancreatic cancer cells [55]. However, as for exosomes from BM-MSCs, they also serve as good carriers. Due to the ability to transfer active molecules (cytokines, growth factors, RNA), it was reported that they were used for the delivery of doxorubicin to breast cancer cells. Researchers demonstrated that with the chimeric LAMP2b-DARP in protein on the surface of exosomes, DOX-exo showed more efficient binding to HER2-positive TUBO cells. The designed exosomes increased the accumulation of DOX in the tumor site and reduce the growth rate of breast cancer cells [56].

Furthermore, some researchers have combined exosomes with other drug delivery systems so that improve the therapeutic effect of EVs. Small extracellular vesicles (sEVs) derived from BM-MSC are widely used in the treatment of myocardial infarction (MI). Researchers designed an alginate brine gel with sEVs (sEVs-gel) in it to increase its retention in the heart in order to improve treatment. Experiments proved that it reduced the apoptosis of cardiomyocytes and promoted the polarization of macrophages. Moreover, the sEVs-gel group had significantly better measurements of cardiac function and infarct area than the sEVs group [57].

To sum up, EVs from BM-MSCs have a wide application in many diseases and have been studied extensively. They showed the potential to be modified or engineered to suit clinical proposes. And they can be also administered in a variety of ways. However, the mechanisms by which it works needs further research.

## EVs from human umbilical cord mesenchymal stem cells (huc-MSCs)

### The therapeutic effects of EVs from huc-MSCs

The human umbilical cord is an excellent source of MSCs, as the human umbilical cord can be largely expanded and has clinical potential in the treatment of some diseases including nerve, cardiovascular, liver, kidney and skin wounds. However, there is growing evidence that EVs from mesenchymal stem cells may contribute to these effects and are considered a potential alternative to stem-based therapies. By exploring the effect and mechanism of hepatic ischemia–reperfusion injury (IRI), it has been revealed that human umbilical cord MSCs-EVs (huc-MSCs-EVs) can reduce the infiltration of neutrophils and reduce the oxidative stress of liver tissue in vivo, so as to protect the liver cell apoptosis induced by IRI to some extent. Further studies showed that the mechanism that EVs reduces oxidative stress is that the enzyme manganese superoxide dismutase (MnSOD) which has anti-apoptosis and anti-oxidation ability is encapsulated in the huc-MSCs-EVs [58]. In addition, huc-MSCs-EVs are proved to promote functional recovery and nerve regeneration in a study of peripheral nerve injury. It promotes the recovery of motor function and the regeneration of axon and reduced gastrocnemius atrophy [59]. Moreover, huc-MSCs-EVs ameliorated bone loss in senile osteoporotic mice. Results showed that the EVs can promote osteogenic differentiation of BMSCs and inhibit osteoclast formation of RAW264.7 cells. Meanwhile, miR-3960 mediates the osteogenesis of BMSCs by huc-MSCs-EVs [60]. At present, it has been reported that EVs ameliorates induced intrauterine adhesions with the combination of estrogen. Whether EVs were administrated alone or in combination with estrogen showed a significant reduction in inflammation and fibrosis. And the combination of EVs and estrogen showed the best therapeutic effect. Therefore, the synergistic effect of huc-MSCs-EVs and estrogen can provide a new strategy for the future clinical combination of drugs [61].

Another type of MSCs is from a peculiar structure in the umbilical cord named Wharton’s Jelly. It is a kind of gelatinous matrix that surrounds and protects the umbilical cord blood vessels [62]. At present, there have been many pieces of research about the plantation of Wharton’s Jelly MSCs(WJMSCs). However, researches on EVs from WJMSCs were fewer. Studies have discovered that Wharton-Jelly MSCs derived EVs had therapeutic effects in renal ischemia/reperfusion injury, which effect is like Wharton-Jelly MSCs [63, 64]. The study reported EVs released from WJMSCs (WJMSC-EVs) can suppress oxidation by enhancing Nrf2/ARE activation [63]. The microvesicles (hWJMSC-MVs) can enhance the proliferation and alleviate the apoptosis of renal cells. Further research showed that they also reduced the macrophages and inhibit inflammation by down-regulating CX3CL1 [64]. Another research also showed this immune function in experimental bronchopulmonary dysplasia [65]. Besides, they have neuron protective effects in perinatal brain injury through anti-inflammation [66, 67]. Therefore, it can be seen that WJMSC-EVs has wide application foreground and potential in the field.

In several studies comparing umbilical cord and bone marrow derived mesenchymal stem cell EVs, these two types of EVs were nearly identical in their physical properties and cargo content, especially for transcripts involved in immunomodulation and proliferation. The obvious difference is that Huc-MSCs produce a higher rate of EVs [68]. However, whether the ratio of components of these two types of extracellular vesicles is the same and the effect on their function remains to be further explored.

### The therapeutic effects of exosomes from huc-MSCs

Exosomes derived from Huc-MSCs (HuC-MSC- Ex) have some therapeutic potentials similar to hBM-MSCs-Ex in neural restoration [68], heart repair [69], protection of liver and kidney [70, 71]. Their therapeutic effect on these diseases is related to the function of promoting angiogenesis and reducing apoptosis. For example, Li et al. reported that exosomes derived from UC-MSC (UC-MSC-Ex) can reduce hepatic inflammation and collagen deposition in liver fibrosis induced by carbon tetrachloride (CCl4) [72]. Jiang et al. and Yan et al. further discovered that UC-MSC-Ex has the function of decreasing oxidative stress and apoptosis in CCl4-Induced Liver Injury [73, 74]. At present, researchers have developed functional peptide hydrogel coated exosomes that derived from Huc-MSCs to promote heart repair. The peptide PA-GHRPS can protect H9C2 cells from H2O2-induced oxidative stress. And PGN hydrogel mixed with it and another peptide can effectively encapsulate exosomes, ensuring the stability and continuous release of exosomes. The results showed that the functional exosomes reduced inflammation, fibrosis and apoptosis, promoted angiogenesis and improved myocardial function [75]. HuC-MSC- Ex have a unique application for perinatal diseases and neonates. A study of perinatal brain injury showed that intranasally administered exosomes played a neuroprotective role in the injury. They induced a significant reduction in specific neuron cell death and promoted damaged normal myelin formation [76]. Another study showed that they can reduce nerve inflammation, as they block the degradation and proliferation of the NFκB inhibitor IκBα in response to the stimulation of phosphorylated protein kinase family of molecules LPS [66]. In a study of preterm newborn infants, researchers found that Huc-MSCs-Ex from term newborn infants had an aerobic respiration capacity independent of the entire mitochondria. And there were functional differences between term exosomes and preterm exosomes that preterm exosomes are unable to synthesize ATP. This indicated that premature babies are less able to repair damaged tissue and more able to cope with hypoxia [77]. Additionally, HuC-MSC- Ex had therapeutic effects on ovarian granulosa cell (OGC) apoptosis induced by cisplatin chemotherapy. The exosomes were successfully uptake by OGC and increased the number of living cells. Further evidence suggested that Bcl-2 and caspase-3 expressions are up-regulated, and the expressions of Bax, cleaved caspase-3 and cleaved PARP are down-regulated, indicating the protective effect of exosomes on cells [78].

In addition, some studies have pretreated exosomes such as blue light to investigate the effect on them. Under blue light exposure, HuC-MSC- Ex can promote angiogenesis in vivo and enhance its ability to promote angiogenesis. It was confirmed that the exosomes treated by blue light stimulated the activation of endothelial cells by upregulating the two miRNAs of mir-135b-5p and mir-499a-3p [79].

Compared to other sources of MSCs, exosomes from huc-MSCs have advantages in gynecological and infant diseases, but the reason remains unknown. Additionally, some external stimulation of exosomes may also promote its therapeutic effect.

### The contents in huc-MSCs-Ex related to the effect

It is well known that exosomes contain small molecules that regulate cell function, including RNA and proteins. microRNAs and proteins in exosomes derived from Huc-MSCs have been further studied in many pieces of research. For example, it was found that miR-146a which was an anti-inflammatory miRNA, was strongly upregulated in TNF-α-stimulated Huc-MSCs and was enriched in exosomes. The exosome containing mir-146a was successfully uptake by fibroblasts. And it inhibited fibroblast activation and associated inflammatory responses, which may result in inhibiting urethral stricture [80]. Another research reported that the mir-146a enriched exosomes had a good effect on sepsis. The mir-146a was transferred to macrophages by huc-MSCs-Ex, resulted in M2 polarization. The increased survival in septic mice further demonstrated the efficacy of this exosome. Different from the former, the way to obtain such exosomes was pretreated Huc-MSCs with pro-inflammatory cytokines interleukin-1β (IL-1β) [81]. Moreover, in a study on treating burns, Mir-181c in huc-MSCs-Ex plays a key role in regulating inflammation. They suppressed the increase of levels of tumor necrosis factor cytokines α(TNF-α) and interleukin-1β (IL-1β) and upregulated levels of iL-10. And the over-expression of mir-181c huc-MSCs exosomes can more effectively inhibit the TLR4 signaling pathway and reduce the inflammatory response in burned rats [82]. In addition to promoting wound healing, the research has been done on whether mesenchymal stem cells promote scar formation. The results showed that Huc-MSCs reduced scarring and myofibroblast accumulation in mouse models with skin defects by huc-MSCs-Ex. High throughput RNA sequence and functional analysis revealed that specific microRNAs (Mir-21, -23a, -125b, -145) in exosomes play a key role in inhibiting myofibroblast aggregation which related to factor-β2/SMAD2 pathway [83]. Furthermore, huc-MSCs-Ex contained special protein involved in the protective effect. In previous studies, huc-MSCs-Ex were observed to have antioxidant and anti-apoptotic effects and were rescued from liver failure. A recent study revealed that glutathione peroxidase 1 (GPX1) contained in huc-MSCs-Ex, which detoxifies CCl and HO, can reduce oxidative stress and apoptosis. Therefore, huc-MSCs-ex promotes the recovery of liver oxidative injury by delivering GPX1 [74].

## EVs from adipose tissue-derived mesenchymal stem cells (AD-MSCs)

### The therapeutic effects of EVs from AD-MSCs

As for adipose tissue-derived mesenchymal stem cells, it is made from adipose tissue extracted from healthy adult donors or animals [55]. The main part of extracting is the donor’s abdomen and buttocks. Then special culture medium was used for filtrating MSCs [56].

EVs from human adipose tissue-derived mesenchymal stem cells (AD-MSCs) was shown to be effective in a variety of disease. Most of the functions of hAD-MSCs EVs were basically the same as those of the other two sources, such as tissue repair, reduction of injuries and anti-inflammation. Many studies have further investigated the mechanism by which EVs regulates inflammation in various disease models. In studies of osteoarthritis, EVs from AD-MSCs stimulated with interleukin- (IL-) 1β reduced the production of inflammatory mediators IL-6 and prostaglandin E2 significantly [84]. In another study, it was discovered that AD-MSCs reduced the levels of other inflammatory mediators tumor necrosis factor-α, prostaglandin E2 and NO. And the downregulation of prostaglandin E2 was caused by the reduction of cyclooxygenase-2 and microsomal prostaglandin E synthase-1. Besides, the anti-inflammatory cytokine IL-10 was upregulated, which also indicated that AD-MSCs EVs have anti-inflammatory effects [85]. Further research also showed that MMP3 was downregulated and CCL2 and CCL5 returned at pre-inflammation basal levels. CXCL8 showed significant contractions, suggesting that EVs regulates inflammation through the CREB pathway. In summary, AD-MSCs EVs decreased the expression of pro-inflammatory cytokines and chemokines in the chronic FLS inflammatory model [86]. Moreover, AD-MSCs EVs down-regulated β-galactosidase activity and the accumulation of γH2AX foci which was associated with senescence. Meanwhile, they also controlled the alteration of mitochondrial membrane potential and reduced the level of oxidative stress [84]. The anti-inflammatory effects of AD-MSCs EVs were also found in allergic asthma, with the reduction of IL-5 levels in lung tissue. However, AD-MSCs or EVs had different effects on eosinophil cell counts, levels of IL-4, IL-13, and eotaxin in lung tissue, indicating that the mechanism of anti-inflammation might be different [87].

EVs from murine AD-MSCs have also been shown to reduce neuroinflammation. Prophylactic intravenous injection of AD-MSCs nanovesicles which was 40-100 nm diameter extracellular vesicles, prior to onset significantly reduced experimental autoimmune encephalomyelitis (EAE) severity, reduced spinal cord inflammation, and demyelination [88]. And Low doses of EVs protect neurons from apoptotic cell death [89].

Studies have shown that there were differences in uptake efficiency between the EVs of BM-MSCs and AD-MSCs, but the reason for this phenomenon has not been found yet [90].

### The therapeutic effects of exosomes from AD-MSCs

Exosomes derived from AD-MSCs have similar functions to other sources of exosomes, such as anti-inflammation. In a study of atopic dermatitis, AD-MSCs exosomes significantly reduced mRNA expression of various inflammatory cytokines such as interleukin (IL)-4, IL-23, IL-31, and tumor necrosis factor-α (TNF-α) in atopic dermatitis skin lesions of the mouse model [91]. The decreased inflammatory factors overlapped with those of exosomes from other sources. AD-MSCs exosomes can also modulate the polarization of macrophages by delivering miRNAs [92]. In the aspect of wound healing, AD-MSCs exosomes were absorbed and internalized by fibroblasts and increased gene expression of N-cadherin, cyclin-1, PCNA and collagen I, III. Further research found that in the early stage of wound healing, systemic administration of AD-MSCs exosomes can increase the production of type I and type III collagen. However, at the late stage of wound healing, exosomes may inhibit collagen expression to reduce scar formation [93]. Another study pointed out that AD-MSCs exosomes reduced scar formation by adjusting the ratio of collagen type III and type I [94]. At present, engineering bioactive antibacterial exosomes hydrogel named FHE@exosomes have been developed for wound therapy. The hydrogel was mainly composed of polypeptide and oxidative hyaluronic acid (OHA), which had thermal-responsive property. The hydrogel regulated the release of exosomes. Results showed that this FHE@exosomes had rapid self—healing and high antibacterial activity [95]. In addition, AD-MSCs exosomes with miR-375-overexpressing promoted bone regeneration [96]. This means that when exosomes from BM-MSCs are not suitable for treating the disease, AD-MSCs derived exosomes may be a good alternative. A recent study tried to combine AD-MSCs exosomes with a polylactic acid-glycolic acid (PLGA) scaffold to repair the skull defect and obtained certain curative effects [97].

Previous studies reported that transplanting AD-MSCs with miR-122 modification suppressed liver fibrosis [98]. Therefore, it was investigated that the therapeutic effect of exosomes derived from miR-122 modified AD-MSCs against hepatocellular carcinoma. Results showed that exosomes successfully transferred miR-122 to hepatocellular carcinoma cells and enhanced chemical sensitivity [99]. This means AD-MSCs exosomes have the potential for adjuvant chemotherapy.

Exosomes from ADSCs works in a wide and dispersed range of diseases [100], and a growing number of studies have shown that it has similar effects to exosomes from other sources of MSCs. It indicated that AD-MSCs exosomes can be an alternative when exosomes from other sources have difficulties to extract or are not suitable for therapy. However, researches of AD-MSCs exosomes are still in an early stage and therapeutic functions need to be explored further. Additionally, this source has advantages in its high availability. Therefore, exosomes from this source have a broad application prospect.

## EVs from human embryonic mesenchymal stem cells (ES-MSCs)

Human embryonic mesenchymal stem cells are MSCs formed by the special induced differentiation of embryonic stem cells [101]. The proliferation and differentiation ability of ES-MSCs is weaker than that of embryonic stem cells after differentiation. Because adult mesenchymal stem cells (MSCs) exist in many tissues of the human body but are scarce and lack the pluripotent differentiation ability of embryonic stem cells, the embryonic mesenchymal stem cells have the irreplaceable advantages of the above three types of MSCs [102]. In addition, Studies showed that ES-MSCs have better immunomodulatory activity compared to BM- and AD-MSCs [103]. However, ethical issues may limit their application. And, like mesenchymal stem cells from other sources, cell rejection, ectopic tissue formation, and infusion toxicity cannot be ignored. EVs derived from ES-MSCs avoid most of the above risks and retain similar advantages to the parental MSCs.

At present, researches on ES-MSCs EVs are still at an early stage. The therapeutic effect of ES-MSCs EVs to various diseases need to explore. In a study of liver injury, Mardpour et al. showed that ES-MSCs EVs reduces liver fibrosis and collagen density. Further researches have shown that EVs upregulated apoptotic gene (BCL-2) and anti-inflammatory cytokines (TGF-β1 and IL-10) and showed immunoregulatory activity comparable to that of parental cells. These results indicated that ES-MSCs EVs ameliorated cirrhosis effectively [103]. Due to the anti-inflammatory effect, ES-MSCs EVs may have the potential therapeutic function to a variety of diseases, including allogenic skin grafts, cartilage repair and cerebral ischemia [104–106]. For ES-MSCs exosomes, studies revealed that they have a therapeutic effect on osteoarthritis and osteochondral defect [107, 108]. It was observed that the cartilage defect treated by ES-MSCs exosomes appeared well-formed hyaline cartilage, which completely binds to adjacent cartilage and extracellular matrix deposition that is very similar to age-matched unoperated controls [108]. Another study of osteoarthritis, exosomes from ES-MSCs promotes chondrocyte proliferation and collagen type II synthesis. Besides, they reduced the expression of inflammatory cytokines IL-1β [107].

Human embryonic mesenchymal stem cells have incomparable advantages over other sources, that is, longer telomeres, fast growth, a wider differentiation potential than adult MSCs [106]. However, Whether EVs derived from ES-MSCs are more potent in promoting proliferation and differentiation than adult MSCs remains to be further studied.

### Clinical trials of the four sources of MSCs-EVs

At present, some translations of MSCs-EV based therapy entered into clinical practice (Table 1) and the data is obtained from https://clinicaltrials.gov/. Clinical trials of EVs from UC-MSCs are the most, including chronic kidney disease, macular degeneration, dry eye, and diabetes mellitus. In the trials of kidney diseases, the improvement of renal function was observed, indicating the promising clinical potential of UC-MSCs [109]. In another study of diabetes mellitus type 1 which was in phase 3, the results remained unknown. Two trials related to eye diseases are in recruiting. One ongoing trial is aimed at the dry eye in patients with cGVHD. The treatment group will receive artificial tears for 2 weeks to normalize the baseline, before using the exosomes derived from UC-MSCs. The other is to assess the safety and efficacy of exosomes from UC-MSC for promoting the healing of large and refractory macular holes. The research is in early phase 1. Currently, there are two clinical trials of EVs derived from BM-MSCs. One research is to assess the safety and efficacy of Intravenous Infusion of BM-MSCs derived EVs in preterm neonates at high risk for bronchopulmonary dysplasia. The study is in recruiting. And the clinic trial of inhalation of BM-MSCs derived exosomes to treat coronavirus is not recruiting yet. For clinical researches of AD—MSCs, they are still testing secretome from AD –MSCs, which contains many EVs. However, the effect that secretome exert doesn’t mean that it is due to EVs from AD-MSCs. Besides, there is no clinical trial of EVs from ES-MSCs, which also indicates the research of this source is still in an early stage. Above all, the current situation of clinical trials shows that BM-MSCs and UC-MSCs will be converted into clinical applications more quickly than the other two sources.

**Table 1:** The clinical trials of MSCs-EVs

Sources	Diseases	Intervention	N. Pats	Follow up	State	Location	Number/Ref.
BM-MSCs	Bronchopulmo-nary Dysplasia	Bone marrow mesenchymal stem cell-derived extracellular vesicles	18	40 weeks	Recruiting	Boston Children’s Hospital Boston, Massachusetts, United States Brigham and Women’s Hospital Boston, Massachusetts, United States (and 3 more…)	NCT03857841
BM-MSCs	Coronavirus	5 times aerosol inhalation of MSCs-derived exosomes (2.0*10E8 nano vesicles/3 ml at Day 1, Day 2, Day 3, Day 4, Day 5)	30	3.5 months	Not yet recruiting	Ruijin Hospital, shanghai, China	NCT04276987
UC-MSCs	Chronic kidney disease	Umbilical cord MSC-EVs (100 μg/kg/dose)	20	1 year	Concluded	Sahel Teaching Hospital Sahel, Cairo, Egypt	[109]
UC-MSCs	Macular degeneration	Cord tissue MSC-EVs injected directly around macular hole	44	24 weeks	Recruiting	Tianjin Medical University Hospital Tianjin, China	NCT03437759
UC-MSCs	Dry Eye	Umbilical mesenchymal stem cells derived Exosomes	27	12 weeks	Recruiting	Zhongshan Ophthalmic Center Guangzhou, Guangdong, China	NCT04213248
UC-MSCs	Diabetes mellitus Type 1	Two doses of MSC-EVs	20	3 months	Unknown	Sahel Teaching Hospital Sahel, Cairo, Egypt	NCT02138331
AD-MSCs	Osteoarthritis	Secretome from adipose-derived mesenchymal stromal cells	24	3 years	Not yet recruiting	/	NCT04223622

Although clinical trials of MSCs-EVs have made some progress, there are also challenges for clinical translational studies. First, the side effects of MSCs-EVs in clinical applications are not clear. Besides, they lack standardized manufacturing processes and methods, which need to be solved in the future.

## Discussion

EVs, widely found in body fluids are produced by almost all cell types. Most mature cells produce very little EVs, thus it is hard to translate applications [11]. At present, except for MSCs-EVs, EVs from tumor cells and immune cells have been studied a lot. EVs derived from tumor cells contain RNA and other content that may promote cancer [110]. Although it was reported that EVs from tumor cells were used to deliver drugs to target cancer cells [111], the risk of tumor promotion should not be ignored. EVs from immune cells such as dendritic cells and macrophages have also been studied extensively. However, conditions of cell culture are more demanding and it requires technical support related to cell-induced differentiation. Additionally, EVs from immune cells are not enough to activate potent effects in vivo [112]. The technical difficulties for transformation and applications are considerable. MSCs have an excellent capacity for proliferation and can produce a large number of EVs. Meanwhile, their risk of promoting tumor growth and metastasis is less than tumor EVs.

EVs secreted from different MSCs are proved to have effects on cardiac, kidney, liver and brain diseases and the functions have been summarized in Table 2. Their effects bear some similarity to their parental MSCs, for they carried part of the content of the parental cells. Thus, EVs are also regarded as an alternative for cell-free therapy. Compared to using MSCs directly for disease treatment, EVs reduced the risk of rejection of transplantation and variation.

**Table 2:** The therapeutic effects for different MSC-EV type

Origins	Type of vesicles	Diseases	Administration route	Biological function	References
hBM-MSCs	EVs	Osteoarthritis	/	Promoting extracorporeal cartilage regeneration	[26]
hBM-MSCs	EVs	Tendon injury	Topical administration	Promoting tendon healing	[30]
hBM-MSCs	EVs	Kidney injury	Intravenous injection	Repairing kidney damage	[31, 32]
hBM-MSCs	EVs	Graft-versus-host disease	Systemic infusion	Suppressing inflammation	[35]
hBM-MSCs	Exosomes	Liver fibrosis	Intraperitoneal injection	Reducing hepatic fibrosis	[41]
hBM-MSCs	Exosomes	Autism spectrum disorder	Nasal administration	Improving autistic behavior	[42]
mBM-MSCs	EVs	Hepatic failure	Intravenous injection	Tissue regeneration	[37]
mBM-MSCs	EVs	Alzheimer’s Disease	Intracerebral Injection	Reducing a beta plaque	[38]
mBM-MSCs	Exosomes	Heart attacks	Intramyocardial injection	Inducing macrophage polarization	[45]
mBM-MSCs	Exosomes	Experimental autoimmune encephalomyelitis	Intraperitoneal injection	Regulating microglia polarization	[46]
huc-MSCs	EVs	Hepatic ischemia–reperfusion injury	Intraperitoneal injection	Protecting the liver cell apoptosis	[58]
huc-MSCs	EVs	Peripheral nerve injury	Intravenous injection	Promoting nerve regeneration	[59]
huc-MSCs	EVs	Senile osteoporosis	Intravenous injection	Ameliorating bone loss	[60]
huc-MSCs	EVs	Intrauterine adhesions	Intravenous injection	Ameliorating induced intrauterine adhesions	[61]
WJMSCs	EVs microvesicles	kidney injury	Intravenous injection	Suppressing oxidation alleviating the apoptosis of renal cells	[63] [64]
WJMSC	EVs	Perinatal brain injury	Intraperitoneal injection	Suppressing inflammation	[67]
huc-MSCs	Exosomes	Liver injury	/	Promoting angiogenesis and reducing apoptosis	[72–74]
huC-MSC	Exosomes	Perinatal diseases and neonates	Intranasal administration	Protecting neuron cell	[76]
huC-MSC	Exosomes	Preterm newborn infants	/	Increasing capability to cope with anoxic environment	[77]
huC-MSC	Exosomes	Primary ovarian insufficiency (poi)	/	Preventing and treating chemotherapy-induced ovarian granulosa cell apoptosis	[78]
hAD-MSCs	EVs	Osteoarthritis experimental allergic asthma	/	Regulating inflammation	[84, 85, 87]
mAD-MSCs	EVs	Experimental autoimmune encephalomyelitis	Intravenous injection	Reducing neuroinflammation	[88]
AD-MSCs	Exosomes	Atopic dermatitis	Intravenous injection or subcutaneously injection	Regulating inflammation and modulating the polarization of macrophage	[91]
AD-MSCs	Exosomes	Wound healing	Systemic administration	Reducing scar formation	[93, 94]
ES-MSCs	EVs	Liver fibrosis	Intraperitoneal injection	Ameliorating rat liver fibrosis	[103]
ES-MSCs	Exosomes	Osteoarthritis	Intra-articular injections	Promoting chondrocyte proliferation	[107, 108]

Although the origins of MSC-EVs are different, they have something in common. EVs derived from different sources of MSCs all have promoting angiogenesis effect. And EVs from bone marrow MSCs, human umbilical cord MSCs and human embryonic MSCs can enhance tissue repair. Except for EVs from BM-MSCs, three other kinds of MSC-EVs can suppress inflammation, but the mechanisms are a little bit different.

One of these four MSCs-EVs will show a better therapeutic effect than other kinds of EVs in certain diseases. For neurodegenerative pathologies, menstrual Mesenchymal stem cells derived exosomes enhanced neuritic outgrowth most among 4 sources MSC exosomes, including menstrual, umbilical cord, bone marrow MSCs and chorion stem cells. The research also indicated that microvesicles had opposite effects on neurons, inhibiting the growth of neurons [4]. And for glioblastoma, BM-MSCs EVs and UC-MSC EVs decreased glioblastoma cell proliferation and induced cell apoptosis, while adipose tissue MSC-EVs increased glioblastoma cell proliferation and had no effect with cell apoptosis. Experiments also showed that UC-MSC EVs performed superior effects on glioblastoma and can deliver drugs to cancer cells [21]. As for human embryonic mesenchymal stem cells, they had a more powerful neuroprotective capacity than fetal MSC-EVs [106]. However, for other diseases, which MSCs-EVs are more effective need to be further explored.

Proteomic analysis of EVs also suggested their functions. For EVs from different sources of MSCs, they contain partly similar proteins. Similar proteins account for nearly half of all proteins [113]. Additionally, it was reported that 60% of proteins were the same between EVs from human bone marrow-derived and human umbilical cord blood-derived MSCs, which were associated with cell growth and/or maintenance and anti-oxidative stress [114]. Such function-related proteins were also found in EVs from adipose tissue MSCs [115]. However, whether they are the same proteins is uncertain.

Through proteomic analysis, the mechanism of the therapeutic effect of EVs can be found. And the selective enrichment of protein of different MSCs-derived EVs indicated the difference of functions of EVs from various MSCs and the preference in Proteomic of different EVs. It partly confirms its therapeutic effect. And the effect can be changed through knockdown of specific proteins. Also, the functions of some groups of proteins can be predicted by GO analysis and this may provide directions for further research.

## Conclusion and future perspective

EVs from different sources of MSC has some unique effects in some diseases and need further studies of the mechanisms. In addition, EVs are also a promising carrier, for they have better biocompatibility and intrinsic targeting ability than ordinary nanocarriers such as liposomes. Considering the specific clinical diseases, EVs from different sources can be selected and designed. Apart from exploring continually the clinical applications of MSC-EVs, we need some methods to get a large quantity of stable EVs quickly and identify the side-effect in clinical trials.

## Data Availability

Not applicable.
